# Evaluation of bone mineral density and body compositions interrelation in young and middle-aged male patients with Crohn’s disease by quantitative computed tomography

**DOI:** 10.3389/fendo.2022.953289

**Published:** 2022-09-23

**Authors:** Xueli Zhang, Kun Peng, Gang Li, Lidi Wan, Tingting Xu, Zhijun Cui, Fuxia Xiao, Li Li, Zhanju Liu, Lin Zhang, Guangyu Tang

**Affiliations:** ^1^Department of Radiology, Shanghai Tenth People’s Hospital, School of Medicine, Tongji University, Shanghai, China; ^2^Department of Radiology, Shanghai East Hospital, Tongji University of Medicine, Shanghai, China; ^3^Department of Radiology, Chongming branch of Shanghai Tenth People’s Hospital, Shanghai, China; ^4^Department of Gastroenterology, Shanghai Tenth People’s Hospital, School of Medicine, Tongji University, Shanghai, China

**Keywords:** bone mineral density, body compositions, Crohn’s disease, quantitative computed tomography, bone geometric parameters

## Abstract

**Background:**

The aim of this study was to investigate the characteristics of bone mineral density (BMD) and body compositions, and the impact of body compositions on BMD in young and middle-aged male patients with Crohn’s disease (CD).

**Methods:**

Patients with CD (n = 198) and normal controls (n = 123) underwent quantitative computed tomography (QCT) examination of lumbar vertebrae 1–3 (L1–3). The BMD and bone geometric parameters were measured and outputted by QCT post-process software. Meanwhile, body composition parameters, including subcutaneous adipose tissue (SAT), visceral adipose tissue (VAT), lean mass (LM), and muscles mass around lumbar vertebrae were also acquired by QCT. Blood indicators [interleukin (IL)-6, IL-8, tumor necrosis factor alpha (TNF-α), C-reactive protein (CRP), Ca, and P] were collected from clinical medical records. Independent *t*-test was used to compare these variables between the CD group and the normal control group.

**Results:**

There was no significant difference in age, height, and weight between the CD group and the control group (*p* > 0.05), indicating that the sample size was relatively balanced. Mean BMD in the CD group were lower than those in the control group, but the difference was not statistically significant (*p* > 0.05). The bone geometric parameters of the CD group, including cortical area/density (Ct. Ar, Ct. BMD) and trabecular area/density (Tb. Ar and Tb. BMD), were significantly lower than those of the control group (*p* < 0.05), so were the body composition parameters including total adipose tissue (TAT), visceral adipose tissue (VAT), subcutaneous adipose tissue (SAT), lean mass (LM), and muscles mass (*p* < 0.05). In addition, the level of plasma IL-6, IL-8, CRP, and TNF-α of the CD group were higher than those of the control group (*p* < 0.05). On the contrary, the body mass index (BMI) and serum Ca and P levels of the CD group were lower than those of the control group (*p* < 0.05). Through multiple linear regression analysis, Tb. BMD, VAT, Ct. Ar, LM, Ca, and IL-8 entered the regression model and revealed a significant contribution to BMD.

**Conclusions:**

Patients with CD could suffer from reduction in BMD. However, the parameters of bone geometric parameters are more sensitive and accurate than BMD changes. Among them, Tb. BMD, VAT, Ct. Ar, and LM have significant effects on BMD reduction.

## Introduction

Over the last two decades, there has been a substantial increase in inflammatory bowel disease (IBD) worldwide, especially Crohn’s disease (CD) (1, 2). CD is a chronic inflammatory disease of the gastrointestinal tract that can affect many other organ systems, including the musculoskeletal (3). Musculoskeletal system deficits are the most common extra-intestinal manifestations and complications of CD (4). However, the results of the change characteristics of bone mineral density (BMD) and body composition in CD patients are inconsistent. In particular, the relationship between body composition and BMD and the possible internal mechanism is less clear.

Metabolic bone disease can usually affect up to 80% of sufferers in CD patients and lead to a reduction in bone strength and increase in risk of fracture (5, 6). CD presents multiple threats to bone quality, either directly through the inflammatory disease process or indirectly through malnutrition, physical inactivity, loss of muscle mass and strength, inflammatory factor stimulation, and calcium (Ca)/phosphorus (P) deficiency or the influence of medicines that are used to control the CD symptom, such as glucocorticoids (7). Ward et al. have found that patients diagnosed with CD have low bone and muscle mass (3). Other studies reported body compositions and body mass index (BMI) to be the strongest predictors of BMD in CD patients (8). Low BMI, which can reduce skeletal loading at weight bearing, has consistently been found to be associated with both osteopenia and osteoporosis in CD cohorts. Studies on pediatric and adults with IBD showed that muscle mass and function were lower compared to healthy volunteers (3, 9). Muscle deficits may contribute to overall disease activity and are adversely associated with responsiveness to IBD therapies (10). Some inflammatory mediators or serum concentrations may also contribute to bone loss in CD, such as tumor necrosis factor (TNF-α), interleukin (IL)-6, IL-8, C-reactive protein (CRP), Ca, and P. The mechanism of their effect on musculoskeletal system needs to be further studied.

Osteoporosis is a common age-related disease of abnormal bone metabolism, especially in postmenopausal women (11). Thus, only the young and middle-aged men were included in this study to exclude the effect of age, hormone, and other factors on bone metabolism. A comprehensive examination of musculoskeletal health was performed by quantitative computed tomography (QCT) in male patients with CD. QCT can not only determine the bone geometric parameters including cortical area (Ct. Ar, mm^2^), trabecular area (Tb. Ar, mm^2^), and volumetric BMD (v-BMD) measurements including total BMD (Tt. BMD, mg/cm^3^), trabecular BMD (Tb. BMD, mg/cm^3^), and cortical BMD (Ct. BMD, mg/cm^3^) (12) but also measure body composition parameters, including lean mass (LM), muscles mass, total adipose tissue (TAT), subcutaneous adipose tissue (SAT), and visceral adipose tissue (VAT), which are considered gold standards for assessing adipose and muscle distribution (13–15).

The purpose of this research was to investigate the characteristics of BMD and body composition change in young and middle-aged men with CD by QCT and to evaluate the effect of body compositions, plasma inflammatory factors, and bone metabolic indicators on BMD.

## Material and methods

### Subjects

It is a retrospective study that include 198 young and middle-aged male patients with CD (18–59 years old) from a database of CD patients who underwent computed tomography (CT) examination due to abdominal discomfort at the Shanghai Tenth People’s Hospital, Tongji University School of Medicine from January 2019 to December 2021. The length history of CD was >1 year and <20 years. Meanwhile, 123 healthy male subjects (21–60 years old) with transient abdominal pain or discomfort were enrolled as the normal control group. Participants with hepatic or renal disease, hyperparathyroidism, thyrotoxicosis, hypogonadism, malignant tumor, or medication taken affecting BMD were excluded.

**Figure 1:**
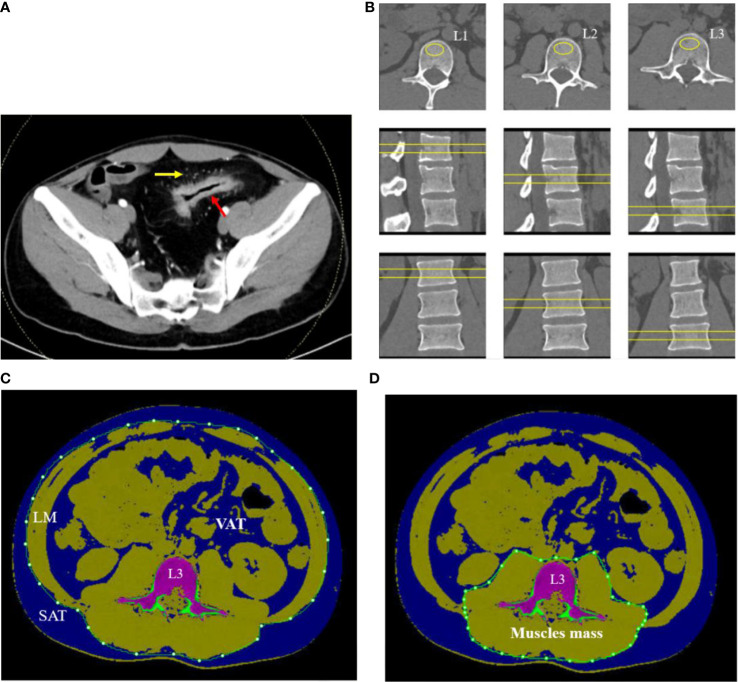
A 32-year-old man with CD. **(A)** Contrast-enhanced CT scan of small intestine showed bowel wall thickening, rich blood supplying (comb sign). **(B)** The L1–L3 BMDs were measured by QCT on axial, coronary, and sagittal plane. **(C)** The SAT, VAT, and LM were manually drawn around the abdominal wall at the level of L3 midplane. **(D)** Muscle mass around lumbar vertebrae were measured within the range manually drawn around the fascial borders of muscles (psoas and paraspinal muscles) at the level of L3.

The diagnosis of CD was based upon the currently accepted criteria (16) and assessed by radiology (**Figure 1A**), histology, and endoscopy. The clinical characteristics of all subjects, including gender, age, height, weight, disease distribution and activity, family history, smoking history, and history of immunosuppressive medication or surgery, were recorded.

### BMD and bone geometric parameters measurement

All subjects underwent dual-source CT (SIEMENS, SOMATOM Force, Germany) examination of the entire abdomen due to abdominal discomfort including the range from lumbar vertebra 1 to 3 (L1–L3). CT examination was performed by the following parameters: tube voltages, 120 kV; tube current, 125 mAs; table height, 120 cm; slice thickness, 1.25 mm; and matrix size, 512 × 512. Meanwhile, without contrast-enhanced CT images were uploaded to QCT workstation and analyzed using the three-dimensional (3D) spine function QCT pro software (Mindways Inc., Austin, TX, USA).

Regions of interest (ROIs) were placed in the central L1–L3 vertebral body on axial, sagittal, and coronal images to measure the mean v-BMD (**Figure 1B**). The area of ROI approximately 250–350 mm^2^ was as large as possible within the boundary of choice. The values of v-BMD of L1, L2, and L3 vertebral body and the bone geometric parameters such as cortical area (Ct. Ar, mm^2^), trabecular area (Tb. Ar, mm^2^), trabecular BMD (Tb. BMD, mg/cm^3^), and cortical BMD (Ct. BMD, mg/cm^3^) were automatically outputted. The mean BMD of the lumbar spine was calculated as the mean value of L1 to L3 vertebral body. Each parameter was measured separately by two radiologists, and the average value of each parameter was taken as the final one.

### Body compositions measurement

On the midsagittal image of L3 vertebral body in CT plain scan of the abdomen, the body composition parameters were obtained with the slice thickness of 1.25 mm using QCT Pro software (Mindways Inc., Austin, TX, USA) and completed synchronously with BMD examination. The voxels within the slice were separated into color-coded objects containing adipose tissue [−190 to −30 Hounsfield units (HU)], muscles (0–100 HU), and bone (≥145 HU) in order to calculate different body compositions easily. A closed contour was drawn around the abdomen and created by the temporarily removal of the skeletal muscle, intestine, vertebra, and others due to the value of density not in the range of the adipose tissue. The subcutaneous adipose tissue (SAT) was defined as one outside of the range manually drawn around the abdominal wall with value of density in the range of adipose tissue (−190 to −30 HU). The visceral adipose tissue (VAT) was defined as the adipose tissue manually within the range around the abdomen. The total adipose tissue (TAT) was computed as the sum of SAT and VAT (**Figure 1C**). The fascial borders of lean mass (LM) were traced manually and were segmented out of the image around the abdomen (**Figure 1C**). Muscle mass around the lumbar vertebrae (psoas and paraspinal muscles) were manually drawn closely along the fascial borders of the muscles (**Figure 1D**). Some automatic identification of nonstandard ROI requires manual adjustment by the radiologists.

### Biochemical assessment

Some serum inflammatory factors and bone metabolic indicators like tumor necrosis factor (TNF-α), interleukin (IL)-6, IL-8, C-reactive protein (CRP), Ca, and P were collected from clinical medical records. The inflammatory mediators of TNF-α, IL-6, and IL-8 were measured by ELISA (BD FACS Canto II, USA). The reference range of IL-6 is 0–5.3 pg/ml, the range of IL-8 is 0–20.6 pg/ml, and the range of TNF-α is 0–4.6 pg/ml. The CRP is measured by immumofluorescence method (i-CHROMA, Korea). The reference range of CRP is <8.20 mg/L. Serum Ca and P levels were measured using an automated chemiluminescence assay (Abbott Alinity c, USA). The reference range of Ca is 2.11–2.52mmol/L, and that of P is 0.85–1.51 mmol/L.

### Statistical analysis

The analyses were performed by SPSS 20.0 (SPSS, Chicago, IL, USA), and statistical analysis was performed by GraphPad Prism 5.0. The statistical description of normally distributed continuous variables was summarized using mean and standard deviation (SD). The variables were compared between the control and CD group using independent *t*-test. A multiple regression analysis was used to evaluate the contribution of body composition parameters and blood parameters to BMD change in the CD group, and the standardized coefficients were reported, with BMD as the dependent variable and body compositions and blood parameters as independent variables (including Ct. Ar, Tb. Ar, Tb. BMD, Ct. BMD, TAT, VAT, LM, IL-6, IL-8, TNF-α, CRP, Ca, and P). *P*-values were two-sided and considered statistically significant at or below a 5% level.

## Results

### Clinical characteristics and blood indicators of participants

Clinical characteristics and blood indicators of participants were summarized (**Table 1**). Age, weight, and height in the CD group were slightly lower than those in the control group; however, no significant difference was obtained between the two groups (*p* > 0.05), indicating that the sample size was relatively balanced. The value of IL-6 and CRP in the CD group were higher than those in the control group; however, there was no significant difference between two groups as well (*p* > 0.05). The values of IL-8 and TNF-α were increased in the CD group compared to those in the control group with significant difference (*p <*0.05). BMI, Ca, and P were decreased in the CD group, compared to those in the control group (*p <*0.05).

**Table 1:** Clinical characteristics and blood indicators of two groups.

Clinical characteristics	Control group^a^ (n=123)	CD group^a^ (n=198)	*p*-Value
**Age**	33.97 ± 14.25	34.57 ± 13.00	0.412
**Weight (kg)**	66.45 ± 9.78	62.77 ± 11.87	0.090
**Height (cm)**	173.89 ± 6.31	172.73 ± 5.71	0.298
**BMI (kg/m^2^)** ^b^	22.48 ± 3.52	20.19 ± 3.36	0.065
**IL-6 (pg/ml)**	32.76 ± 4.58	38.78 ± 4.80	0.102
**IL-8 (pg/ml)** ^b^	227.62 ± 39.56	344.83 ± 53.93	0.019
**TNF-α (pg/ml)** ^b^	4.33 ± 0.85	4.585 ± 0.52	0.003
**CRP (mg/L)**	23.12 ± 6.34	27.60 ± 2.16	0.306
**Ca (mmol/L)** ^b^	2.33 ± 0.04	2.03 ± 0.13	0.000
**P (mmol/L)** ^b^	1.18 ± 0.16	1.14 ± 0.12	0.008

a Mean ± SD.

b There is a significant difference between two groups.

### BMD, bone geometric parameters, and body compositions between two groups

**Figure 2:**
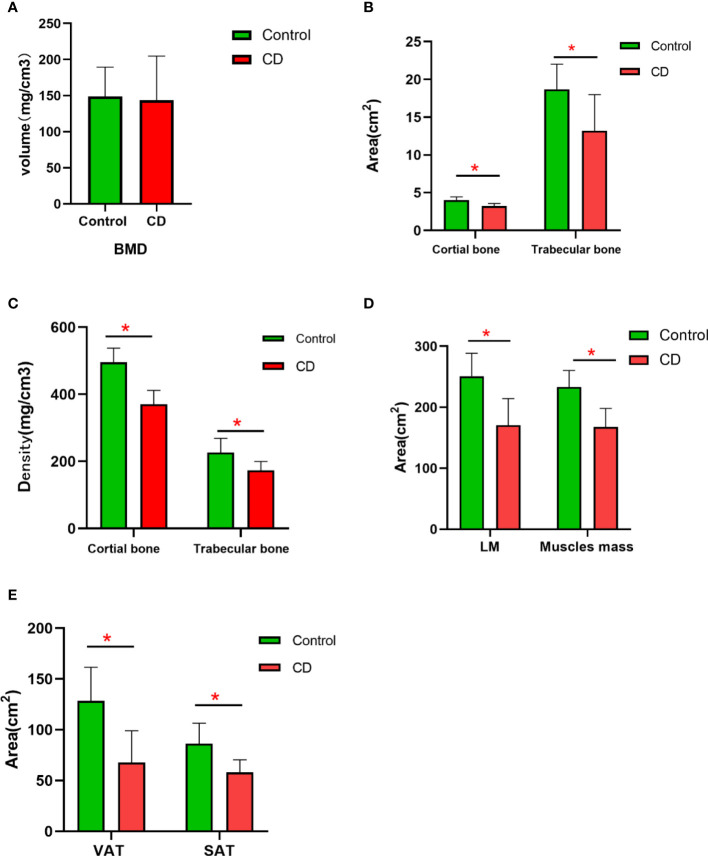
The difference in the variables of BMD and body compositions between control and CD group. **(A)** BMD had no significant difference between two groups (*p* > 0.05). **(B)** The area of the cortical bone and trabecular bone was lower in CD group than those in control group (*p < 0.05). **(C)** The density of the cortical bone and trabecular bone was obviously lower in the CD group than those in control group (*p* < 0.05). **(D)** After adjusting for BMI, the area of LM and muscle mass was lower in the CD group than those in the control group (*p < 0.05). **(E)** After adjusting for BMI, the area of VAT and SAT were obviously lower in the CD group than those in the control group (*p < 0.05).

BMD was decreased in the CD group than those in the control group; however, no significant difference was obtained between the two groups (*p* > 0.05) (**Table 2**, **Figure 2**). The bone geometric parameters such as Ct. Ar, Tb. Ar, Tb. BMD, and Ct. BMD were decreased in the CD group than those in the control group with statistically significant difference (*p <*0.05). After adjusting for the BMI, body composition parameters of TAT, VAT, SAT, LM, and muscles mas around the lumbar spine were decreased in the CD group than those in the control group, and there was statistically significant difference (*p <*0.05).

**Table 2:** The variables of BMD and body compositions were compared between the control and CD group.

Variables	Control group^a^	CD group^a^	*p*-Value
**BMD (mg/cm^3^)**	148.78 ± 40.77	143.84 ± 60.99	0.644
**Ct. Ar (cm^2^)** ^b^	4.01 ± 0.43	3.24 ± 0.34	0. 013
**Tb. Ar (cm^2^)** ^b^	18.69 ± 3.32	13.19 ± 4.79	0.000
**Ct. BMD (mg/cm^3^)** ^b^	495.02 ± 42.48	370.22 ± 41.61	0.000
**Tb. BMD (mg/cm^3^)** ^b^	226.11 ± 42.78	173.49 ± 26.05	0.000
LM (cm^2^)^b*^	250.81 ± 37.70	170.56 ± 43.45	0.000
Muscles mass (cm^2^)^b*^	233.15 ± 27.30	167.69 ± 30.43	0.000
TAT (cm^2^)^b*^	213.31 ± 52.17	139.12 ± 73.44	0.000
VAT (cm^2^)^b*^	128.35 ± 33.21	67.55 ± 31.48	0.000
SAT (cm^2^)^b*^	86.25 ± 20.11	58.13 ± 12.25	0.000

a Mean ± SD.

b There is a significant difference between two groups.

*After adjusting for BMI.

Ct. Ar, cortical area; Tb. Ar, trabecular area; Tb. BMD, trabecular BMD; Ct. BMD, cortical BMD.

### Multiple regression analysis of BMD, body compositions, and blood parameters

BMD is the dependent variable (Y), and body compositions and blood parameters are the independent variables (X1: Ct. Ar; X2: Tb. Ar; X3: Ct. BMD; X4: Tb. BMD; X5: TAT, X6: SAT, X7: VAT, X8: LM, X9: muscles mass, IL-6, IL-8, TNF-α, CRP, Ca, and P as X10-15, respectively). The data are shown in **Table 3**.

**Table 3:** Stepwise multivariate analysis of BMD, body compositions, and blood parameters.

Model		Std *β*	*p-*Value	*F*	*R*^2^
**M1**	**Constant**	−11.019	0.953	79.334	0.486
** **	**Tb. BMD**	0.682	0		
**M2**	**Constant**	36.797	0.059	61.698	0.598
	**Tb. BMD**	0.636	0		
** **	**VAT**	−0.299	0		
**M3**	**Constant**	3.501	0.48	46.159	0.628
	**Tb. BMD**	0.745	0		
** **	**VAT**	−0.243	0		
** **	**Ct. Ar**	0.303	0.012		
**M4**	**Constant**	−108.898	0.015	40.503	0.667
** **	**Tb. BMD**	0.945	0		
**M5**	**VAT**	−0.144	0.002	39.454	0.711
	**Ct. Ar**	0.339	0		
	**LM**	0.301	0.003		
	**Constant**	−87.266	0.039		
	**Tb. BMD**	1.337	0		
	**VAT**	−0.139	0.002		
	**Ct. Ar**	0.411	0		
	**LM**	0.325	0		
	**IL-8**	−0.167	0.001		

Tb. BMD, trabecular BMD; Ct. Ar, cortical area.

Predictive variables (constant): Tb. BMD, VAT, Ct. Ar, LM, and IL-8.

Dependent variable: BMD.

Tb. BMD was the first variable to enter model 1 (M1) and revealed a significant positive contribution to BMD in all models with the greatest *β*-value (*β* = 0.682) in all indices. Then, VAT got into model 2 (M2, *β* = −0. 299), Ct. Ar into model 3 (M3, *β* = 0.303), LM into model 4 (M4, *β* = 0.301), and IL-8 into model 5 (M5, *β* = −0.167). Other indices such as Tb. Ar, Ct. BMD, TAT, and muscle mass had no significant effect on BMD in each model, so they were removed from models and regression equation. The value of *R*^2^ represented the expository power of the model. It showed the highest value in M5 (*R*^2 =^ 0.711), which illustrated that M5 has the best fitting effect on BMD. Finally, a regression equation was obtained: Y=−87.266+1.337X4−0.139X7+0.411X1−0.325X9−0.167X11.

## Discussion

The study investigated the association between BMD and body compositions in young and middle-aged male CD patients and further researched the impact of body compositions on BMD. In many patients with IBD, particularly with CD, the body compositions, reflected by the proportions of bone, adipose tissue, and lean mass, may be abnormal. However, in CD patients, the relationship between body compositions and BMD is complex and controversial. There have been several studies that examined BMD and body compositions in patients with CD by dual energy X-ray absorptiometry (DXA). Tjellesen and his colleagues compared body compositions at the whole-body level in 31 CD patients and intestinal resection of healthy volunteers (17). They reported bone mineral content, BMI, and LM to be decreased in CD patients compared to that in the control group, whereas fat mass expressed as a percentage of body weight was increased in the diseased cohort. DXA is considered the gold standard screening for the detection of osteoporosis. However, recent studies have used the method of CT (obtained for other purposes, like CT examination of IBD) as a surrogate for DXA (18). For example, Pickhardt et al. have found that CT attenuation values of the lumbar spine on scans obtained for other purpose were non-inferior to DXA scans completed within 6 months at screening for osteoporosis (19). Such an approach has the added benefit of detecting subclinical vertebral compression fractures, which are also diagnostic of osteoporosis (20). In this study, we showed that all patients with CD undergo CT scans. Our study utilized QCT to obtain BMD, bone geometric parameters, and body compositions, which enabled us to investigate the characteristics of BMD and body compositions changes, and the impact of body compositions on BMD in male patients with CD that were superior to DXA scans (21). This aspect has not been reported before.

Our study demonstrated a trend of lumbar spine BMD reduction, although no significant difference was obtained, which was similar to those of earlier studies in CD patients. More importantly, our results showed that bone geometric parameters such as Ct. Ar, Tb. Ar, Tb. BMD, and Ct. BMD obviously decreased in CD patients, and these variables showed more significant changes than the BMD value did. It suggested that bone geometric parameters may reflect the bone change more accurately and sensitively than BMD. Currently, little is known regarding the change in geometric parameters in CD population, as no studies using QCT examination have been performed exclusively in this population. HR-pQCT studies reported the deficits in trabecular architecture, volumetric bone density, and cortical geometry in young and middle-aged adults with IBD (22). They were similar to what we currently reported of deficits in cortical and trabecular bone geometry in male adults with CD compared to healthy age- and sex-matched controls by QCT. It indicates that some parameters of bone geometry occur earlier than BMD, which represent the comprehensive changes in various bone structures and mineral deposits. Thus, QCT, which can reflect both bone geometric parameters and bone mass, has obvious advantages over DXA that only reflects bone mass.

In addition, our results also showed an important aspect that the area of LM on the abdomen and muscle mass around the lumbar vertebrae was significantly reduced in CD patients compared to normal contrast after adjusting for the BMI. Depletion of lean body mass is associated with lower quality of life, and higher morbidity and mortality commonly occur as part of the aging process (2, 23). At the same time, loss of muscle mass accelerated the BMD reduction. The mechanism for this result may be that the greater muscle mass and strength provided mechanical loading on the skeleton, which promotes the osteocytes to send a signal that either increases the activity of osteoblasts or decreases the activity of osteoclasts (24). LM deficits may also be associated with demonstrable morbidity, including loss of lean mass and strength, altered energy metabolism, and increased susceptibility to infection. According to the concept of a muscle-bone unit, adaptive bone remodeling that can lead to an increase in bone mass strength is determined primarily by effective strain due to muscle contraction (24). LM exerts an additional influence on the skeleton through regional muscle pull and contributes to the loading of the bone. Other possible mechanism is that these disorders are also a characteristic of malnutrition and chronic intestinal inflammation in CD (2). Moreover, it has been shown that increased muscle mass contributed to the increase in BMD and can reduce the vertebral fracture risk (25).

Due to insufficient nutritional absorption and long-term treatment of diseases, the adipose tissue significantly decreased in CD patients. Similarly, the TAT, VAT, and SAT in the CD group were significantly decreased in our study. Our study was a specially designed such that the adipose tissue is divided into VAT and SAT, which displayed different metabolic and immunological profiles (26, 27). VAT has been particularly related to a proinflammatory state and has been implicated in some gastrointestinal diseases, such as fatty liver, cancers, acute pancreatitis, and CD (28). Inflammatory factors secreted by VAT could increase bone resorption through stimulating osteoclast activity, including TNF-a, IL-6, and IL-8, which destroy bone cells and bone structure (29). These fat-releasing cytokines may also contribute to the debilitating musculoskeletal system observed in IBD patients (30, 31). SAT further exerts mechanical stress on the bone as muscle mass do to increase BMD (32). In CD patients, SAT decreased, which led to the decrease in mechanical stimulation to the lumbar spine, which further led to the decrease in BMD. As we all know, leptin and hormone produced by SAT may increase bone mass *via* stimulating osteoblast activity (33). Consequently, the decrease in SAT may weaken the role of stimulating osteoblast activity.

Serum Ca and P were decreased significantly in the CD group, indicating that bone metabolism disorder occurs in CD patients. Ca, which is stored in the bone tissue and embedded in the protein matrix, is required for normal bone growth and development. Adequate intake of Ca can make the bone to reach its peak and reduce its loss. P is also necessary for the prevention of osteoporosis in CD population. The deficiency of P may affect bone health because of its involvement in organic bone matrix synthesis. Abnormal immune response in CD patients will lead to the release of proinflammatory cytokines, such as TNF⁃α, IL⁃6, and IL⁃8, resulting in intestinal mucosal damage. The activated T cells affect bone metabolism by affecting the expression of RANKL that increases the activity of osteoclasts (34). TNF-α plays an important role in osteoclast formation, which can induce the activation of nuclear factor kappa B (NF-kB) transcription and reduce bone formation by inhibiting osteoblast differentiation, consequently leading to the decrease in BMD. Therefore, TNF-α not only plays an important role in the pathogenesis of CD but also participates in bone metabolism and promotes bone resorption *via* regulating osteoclast activity. This study showed that TNF-α in the CD group was higher than that in the control group, suggesting that osteoporosis induced by intestinal inflammation may be related to abnormal autoimmune regulation.

Limitations of the current study should be considered. First, we did not classify CD patients according to clinical activity, history of drug use, and disease length, as this study included a relatively small sample of CD patients. In the future, more CD patients will be collected and classified for further research. Second, there was no comparison between DXA and QCT to determine which assessment was better. Third, QCT measurement is related to equipment, post-processing software, and scanning conditions, so it is not widely used at present.

An individual’s body compositions are important for bone health. Altered body compositions and BMD in CD patients may impact the course of the illness, the response to CD therapies, prognosis, and quality of life. Multiple factors that include the underlying disease activity, malabsorption of Ca and P, and use of glucocorticoids also can influence musculoskeletal outcomes by diverse mechanisms in adults with CD. Many patients control well with CD symptoms; however, there have been persistent deficits in muscles and bone health in published cohorts. The persistence of musculoskeletal deficits is a bad factor for young men with CD. Except for the management and pharmacological therapies on CD disease, it highlights that the effective therapeutic interventions on musculoskeletal health are also very important, which may include nutritional, lifestyle, and pharmacological approaches. For the early detection and treatment evaluation of bone health abnormalities in CD patients, QCT is an effective and convenient examination technology, especially when it is grafted into routine abdominal CT examination.

## Conclusions

The present research suggested that patients with CD could suffer from reduction in BMD; however, the bone geometric parameters were more accurate and sensitive than BMD changes. SAT and muscle mass decreased significantly in CD patients, which further promoted the decrease in BMD. The treatment plan of CD patients must pay attention to the protection of bone health. QCT is an important examination method for the quantitative evaluation of bone metabolism and body composition, which is better to be grafted when abdominal CT examination is performed.

## Data availability statement

The original contributions presented in the study are included in the article/supplementary material. Further inquiries can be directed to the corresponding authors.

## Ethics statement

This study was reviewed and approved by Shanghai Tenth People’s Hospital, Tongji University School of Medicine. The patients/participants provided their written informed consent to participate in this study.

## Author contributions

ZL, LZ, and GT designed the research. XZ, KP, and LW wrote the article. XZ, TX, and LL performed the research. XZ, KP, GL, and ZC collected and analyzed the data. GL, FX, and ZC performed the statistical analysis. ZL, LW, and LZ revised the article. All authors had full access to all of the data in the study, reviewed and edited the manuscript for intellectual content, and had final responsibility for the decision to submit for publication.

## Funding

This work was supported by the National Natural Science Foundation of China (Grant no. 81871325), and Science and technology innovation action project of STCSM (Grant no. 20Y11911800) and Project of Chongming STCSM[CKY2021-41].

## Acknowledgments

The authors wish to thank the patients and volunteers who participated in the study and all the investigators and gastroenterologists of all participating study.

## Conflict of interest

The authors declare that the research was conducted in the absence of any commercial or financial relationships that could be construed as a potential conflict of interest.

## Publisher’s note

All claims expressed in this article are solely those of the authors and do not necessarily represent those of their affiliated organizations, or those of the publisher, the editors and the reviewers. Any product that may be evaluated in this article, or claim that may be made by its manufacturer, is not guaranteed or endorsed by the publisher.
